# Alcohol and Nicotine Use among Adolescents: An Observational Study in a Sicilian Cohort of High School Students

**DOI:** 10.3390/ijerph19106152

**Published:** 2022-05-18

**Authors:** Emanuele Cannizzaro, Gianluca Lavanco, Valentina Castelli, Luigi Cirrincione, Danila Di Majo, Francesco Martines, Antonina Argo, Fulvio Plescia

**Affiliations:** 1 Department of Health Promotion Sciences, Maternal and Child Care, Internal Medicine and Medical Specialties “Giuseppe D’Alessandro”, University of Palermo, Via del Vespro 133, 90127 Palermo, Italy; emanuele.cannizzaro@unipa.it (E.C.); or gianluca.lavanco@unipa.it (G.L.); luigi.cirrincione@unipa.it (L.C.); antonella.argo@unipa.it (A.A.); 2 Department of Biomedicine, Neuroscience and Advanced Diagnostics (BiND), University of Palermo, Via del Vespro 129, 90127 Palermo, Italy; valentina.castelli02@unipa.it (V.C.); danila.dimajo@unipa.it (D.D.M.); francesco.martines@unipa.it (F.M.)

**Keywords:** alcohol, nicotine use, adolescence, binge drinking, nicotine dependence

## Abstract

In recent years, the mode of alcoholic intake known as binge drinking (BD) has become a common practice, especially among adolescents who, due to socio-environmental motives, tend to reach a rapid state of drunkenness. This drunkeness leads to alterations in brain areas responsible for executive functions and cognitive processes, as well as to the genesis of factors that predispose to lasting addiction. Likewise, nicotine leads to a comparable degree of addiction. On this basis, the aim of this research was to evaluate, on a cohort of 349 high school students (15–17 years old) in the province of Palermo, the following: (I) the drinking model of alcoholic beverages; (II) the use of nicotine and the degree of dependence; (III) the correlation between the consumption of alcoholic beverages and the use of nicotine. We employed the AUDIT-C test and the Fagerström test, two valid and standard instruments, in order to assess alcohol and nicotine use, respectively. Statistical analysis of the data showed that male and female students consumed alcohol prominently in a BD mode (77.2%, audit score (AS) 3.497, confidence interval (CI) 3.206–3.788; 69.6%, AS 2.793, CI 2.412–3.274) and nicotine (41.5%, Fagerström score (FS) 3.882, CI 3.519–4.245; 28%, FS 3.286, CI 2.547–4.024). Furthermore, a positive correlation between alcohol consumption and nicotine use was found for male (r = 0.6798, *p* < 0.0001) and female (r = 0.6572, *p* < 0.0001) students. This study provided further insights into the use of legal substances of abuse in adolescents, evidencing the obvious need for the promotion of specific school educational programs aimed at the wellbeing of youth populations.

## 1. Introduction

Alcohol is one of the substances of abuse most consumed by the world’s population. Data on its use show that about 2.3 billion people consume alcoholic beverages, and, among the regions of the World Health Organization, Europe appears as the continent with the highest levels of consumption per capita [1]. Moreover, although it is fading, a traditional difference in the pattern of consumption and type of alcoholic beverages consumed among regions exists [2,3,4]. According to the Italian Institute of Statistics (ISTAT), 77.2% of males and 56.5% of females consume alcohol, and this percentage is continuously increasing [5].

Interestingly, as a result of the COVID-19 pandemic, there has been a recent increase in sales of alcoholic beverages, paralleled with higher alcohol consumption, compared to the pre-pandemic period [6,7,8]. This increase was likely attributable to social isolation and stress, which predisposes people to alcohol consumption [9,10]. Moreover, when an individual is subjected to particularly stressful conditions, he or she tends to consume large amounts of alcohol, which can promote the onset of different disorders related to its use/abuse [11,12,13].

In addition to the increase in the consumption of alcoholic beverages, it is important to note that there was a substantial change in the way alcoholic beverages were consumed. Specifically, high-risk alcohol consumption such as binge drinking (BD)—a drinking pattern that results in a blood alcohol concentration of 0.08 g/dL, due to the ingestion of four alcoholic beverages for women and five for men in a two-hour time period—[14,15], turned out to be most prevalent, rather than low-risk alcohol consumption (one or two alcoholic beverages). Specifically, this highly risky mode of alcohol intake is undertaken by millions of consumers, with a high proportion of individuals between the ages of 11 and 21 years [16,17], the pre- to late-adolescent life span [18,19]. Interestingly, a study conducted on students of the University of Palermo, showed that about 19% of those interviewed experienced risky alcohol consumption [20].

The prevalence of BD among youth is a concern that should not be underestimated because of the short- and long-term health problems that this mode of consumption can cause, such as depression and social rejection [21,22]. In addition, BD has been shown to be a predisposing factor for the use of other substances of abuse including nicotine and marijuana [23,24]. Furthermore, alcohol intake during adolescence is a determinant factor in the genesis of different alcohol-related problems [25] and a predisposing factor for the onset of alcohol abuse or dependence in adulthood [26,27]. Studies conducted in the animal model have highlighted how BD is able to induce structural changes in brain areas that control executive functions and learning and memory processes, such as the prefrontal cortex, hippocampus, and limbic system [28,29].

In addition to alcohol, one of the substances of abuse most consumed by young people is nicotine, a substance present in different concentrations in tobacco or electronic cigarettes [30,31]. In this regard, it is interesting to note that although it is known that tobacco use is responsible for 25% of cancer deaths worldwide and increases the risk of cardiovascular and pulmonary diseases, there is still a high percentage of adolescents (18.4%) who smoke [32]. A study by Health Behaviour in School-aged Children, involving students between 11 and 15 years of age, showed that the percentage of participants who reported having smoked increased with age, both in males (24.8%) and females (31.9%).

As adolescents respond to nicotine differently than adults, they are more vulnerable to the harmful effects caused by its exposure [33]. Furthermore, the younger the age of a person at smoking initiation, the greater the likelihood is of them becoming addicted to tobacco in adulthood [34].

From the above, it is evident that both alcohol and nicotine consumption are among the preferred activities for young adults. This is probably due to the ease with which these substances can be found and to the positive connotation that their consumption assumes among the population of teenagers.

On this basis, the aim of the present research, conducted on first-year high school students from the province of Palermo, was to evaluate the following: the pattern of alcoholic beverage consumption, and the presence of disorders related to its abuse, through the administration of the AUDIT-C test; nicotine consumption and the degree of dependence on it through the administration of the Fagerström test, a standard instrument for assessing the intensity of physical addiction to nicotine. In addition, the correlation between the consumption of alcoholic beverages and the use of nicotine was assessed.

Given the scientific evidence about the increasing consumption of legal substances of abuse among adolescents, the hypothesis of our study was that BD is the most-engaged pattern of alcohol consumption along with nicotine dependence. Furthermore, we hypothesized that nicotine use increases alcohol use and vice versa.

## 2. Materials and Methods

### 2.1. Experimental Design

Data were collected from high schools in the city of Palermo, Italy that were representative of a peripherical area of the city. Students of both sexes were enrolled, aged between 15 and 17 years.

Excluded from the study were all those who, at the time the questionnaires were administered, were over 17 years old, had repeated the same year of high school for more than two years, had been resident in Italy for less than 10 years, and all those who had, according to their teachers, obvious disciplinary problems. All students with a support teacher and those who had obvious health problems were also excluded from the study.

Those who did not fall within the above exclusion criteria were considered eligible for testing.

**Figure 1:**
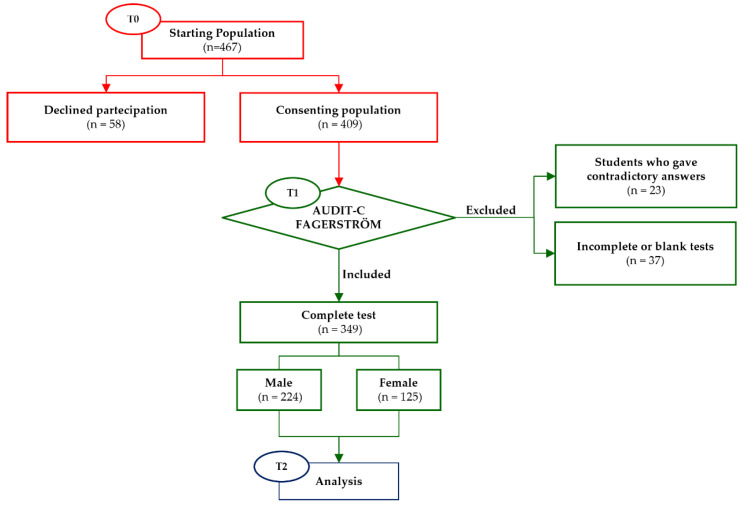
The flowchart of experimental procedures showing the starting population, the exclusion criteria, and the final population that completed the study.

On the day of questionnaire administration, the enrolled population consisted of 467 students. Of these, 12.4% (*n* = 58) decided not to participate and 7.9% (*n* = 37) were excluded because their questionnaires were incomplete or left blank at the time of analysis. A further 4.9% (*n* = 23) were disregarded as they gave contradictory answers to the tests (some participants stated that they did not drink or smoke and then later claimed to take alcoholic beverages or smoke). In the end, the data in the present study related to the analysis of tests correctly completed by 349 first-year high school students: 224 males and 125 females (M/F ratio 1.8) (Table 1) (Figure 1). All participants belonged to a low-middle socioeconomic class, residing mainly in suburban areas of the city.

All the participants, once informed about the purpose of the study, were asked to fill out an informed consent and questionnaires regarding the use of alcoholic beverages and cigarette smoking and to answer simple questions regarding the level of family education and the practice of sports activities.

Completion of the tests was anonymous and voluntary. On the day the questionnaires were administered, a group of researchers went to the schools and, once the questionnaires were completed, proceeded to collect them. The questionnaires were then analyzed by a multidisciplinary group of health experts, which was different from the group that had administered the tests and collected the data.

All data were managed according to the Italian law for the protection of privacy (Decree number 196, January 2003).

### 2.2. Assessment of Alcohol Consumption

Alcohol use was assessed using a short form of the Alcohol Use Disorders Identification Test-Concise (AUDIT-C), a modified version of the ten-question Alcohol Use Disorders Identification Test (AUDIT) developed by the World Health Organization. This test is a brief self-reported alcohol-screening test that is effective for assessing unhealthy alcohol use [35]. This instrument is a three-item survey with a total score ranging from 0 to 12 points. Each item has five response options rated from 0 to 4 points. A score of 3 or more on the AUDIT-C could indicate people who are at-risk drinkers or have alcohol use disorders. A score of 4 or more for men and 3 or more for women is considered predictive of potential alcohol abuse. Commonly, the likelihood of a person having an alcohol use disorder is directly proportional to the highest score on the test.

Furthermore, through the analysis of the individual answers to the questions of the AUDIT-C, this test also allowed us to investigate the following: the frequency with which alcohol was consumed (analysis of the answers given to the first question) and the pattern of alcohol consumption (analysis of the answers given to the second and third questions). Previous studies have confirmed appropriate psychometrics (i.e., Caputo, 2020) [36].

### 2.3. Assessment of Nicotine Dependence

The assessment of nicotine dependence was conducted through the administration of the Fagerström Test for Nicotine Dependence (FTND), a standard instrument that can assess the intensity of physical addiction to nicotine [37]. This test was designed to provide an ordinal measure of nicotine dependence related to cigarette smoking. This instrument consists of six items that are useful for evaluating cigarette consumption, the compulsion to use, and dependence.

In scoring the FTND, yes/no items are scored from 0 to 1 and multiple-choice items are scored from 0 to 3. The items are summed to yield a total score in the range 0–10. The total score was interpreted as follows: mild degree of dependence (0–2); medium degree of dependence (3–4); severe degree of dependence (5–6); and very severe dependence (7–10). The test has previously shown adequate psychometric properties [38].

### 2.4. Statistical Analysis

Statistical analysis of the data was conducted using the GraphPadPrism 8.01 statistical software package (GrapPad Company, San Diego, CA, USA). All data were analyzed using the normal distribution using the D’Agostino–Pearson omnibus normality test in order to determine which statistical tests to apply. The nonparametric Chi-square test was used for the analysis of data that did not have a normal distribution in order to test whether the frequency values obtained by detection were significantly different from the frequencies obtained with the theoretical distribution. The Chi-square test was performed to assess whether there were any differences in the scores obtained from the analysis of the AUDIT-C and FTND between males and females.

A descriptive analysis was also conducted to assess the pattern of alcohol consumption and the degree of nicotine dependence.

The correlation between the AUDIT-C score and FTND was assessed using Pearson’s correlation coefficient test. Simple linear regression analyses were generated as predictive models to assign the correlation found.

## 3. Results

### 3.1. Pattern of Alcohol Consumption

The assessment of the pattern and frequency of alcohol consumption in our sample was carried out through the analysis of the AUDIT-C test.

In detail, among male students (MSs) (224), 173 (77.2%; audit score (AS) 3.497, confidence interval (CI) 3.206–3.788) reported using alcoholic beverages. Among female students (FSs) (125), 87 (69.6%; AS 2.793, CI 2.412–3.274) reported to consume alcohol. Notably, there were no significant differences between MSs and FSs in the percentage of participants consuming alcoholic beverages (χ2 = 2.460, z = 1.568, *p* = 0.1168), drinking occasionally (χ2 = 2.820, z = 1.679, *p* = 0.0931), monthly (χ2 = 0.1116, z = 0.3341, *p* = 0.7383), or weekly (χ2 = 2.031, z = 0.1541, *p* = 0.1541).

According to the AS, the descriptive analysis of the data highlighted a worrying percentage of MSs and FSs with problems related to the consumption of alcoholic beverages and, moreover, that the majority of students of both sexes prefered a binge-like mode of intake (Table 2).

In addition, no statistically significant differences were revealed between MSs with alcohol-related problems (χ2 = 2.143, z = 1.464, *p* = 0.1432) and binge drinking (χ2 = 1.063, z = 1.031, *p* = 0.3025), compared to FSs.

### 3.2. Pattern of Nicotine Consumption

**Figure 2:**
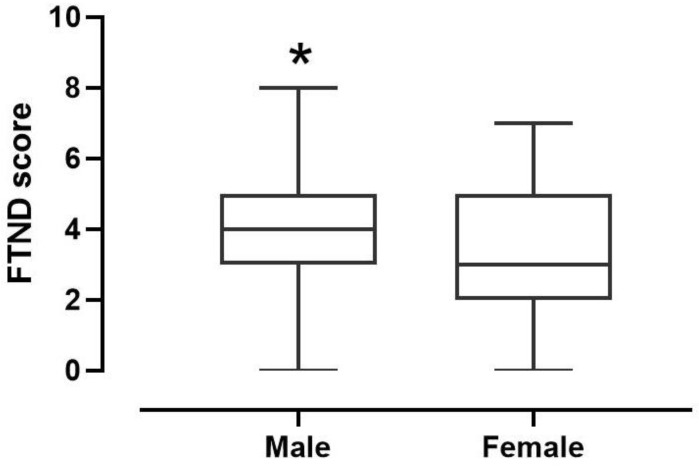
Differences in nicotine consumption between male and female students. * *p* = 0.0120 vs. Female.

The descriptive analysis of the data obtained through the FTND test showed that 93 MSs (41.5%; FTND score (FS) 3.882, confidence interval (CI) 3.519–4.245) and 35 FSs (28%; (FS) 3.286, confidence interval (CI) 2.547–4.024) reported to regularly take nicotine. Notably, there was a statistically significant difference between the percentage of MSs (χ2 = 6.312, z = 2.512, *p* = 0.0120) consuming nicotine compared to that of FSs (Figure 2). Moreover, the descriptive analysis showed a higher prevalence in the severe degree of nicotine dependence in MSs. On the contrary, there was no significant prevalence of degrees of nicotine dependence in FSs (Table 3).

In order to understand whether there were differences in the various degrees of dependence among students of both sexes we conducted the Chi-square test, taking into account the levels of dependence obtained from the FTND analysis. Data analysis did not reveal statistically significant differences in the degree of mild (χ2 = 1.528, z = 1.236, *p* = 0.2164), moderate (χ2 = 0. 0041, z = 0.0065, *p* = 0.946), severe (χ2 = 1.529, z = 1.237, *p* = 0.2163), and really severe (χ2 = 0.9869, z = 0.9935, *p* = 0.3205) cases between MSs and FSs.

### 3.3. Correlation between Alcohol and Nicotine Consumption

**Figure 3:**
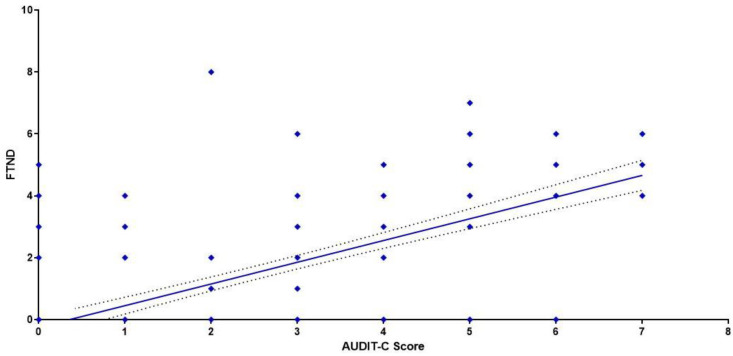
Correlation between AUDIT-C and FTND. Each value represents the mean of 224 male students.

**Figure 4:**
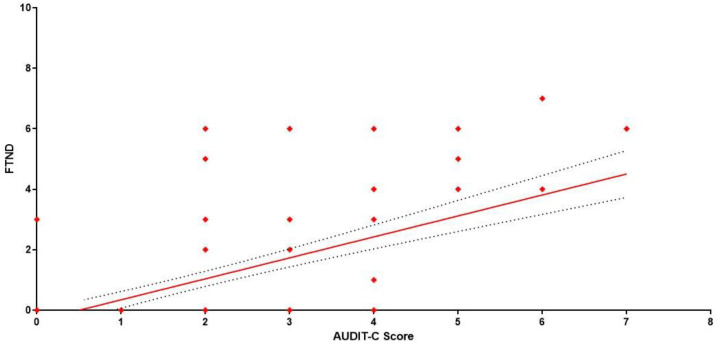
Correlation between AUDIT-C and FTND. Each value represents the mean of 125 female students.

The correlation between alcohol and nicotine consumption was assessed through Pearson’s correlation coefficients between the AUDIT-C score and FTND score: a significant positive correlation was found in MSs (r = 0.6798, CI 0.6024–0.7445, *p* < 0.0001) (Figure 3) and FSs (r =0.6572, CI 0.5444–0.7466, *p* < 0.0001) (Figure 4).

## 4. Discussion

The present observational study, conducted on a population of first-year high school students from a professional institute in the city of Palermo, brought out different alarming trends among adolescents aged between 15 and 17 years. Among the participants our data revealed a high percentage of students who consumed alcoholic beverages (74.5%) and, contextually, nicotine (33.8%).

The detailed analysis of drinking behavior, obtained through the administration of the AUDIT-C, shows differences in the time and mode through which alcoholic beverages are consumed, as well as in the number of participants who present alcohol-related consequences. Indeed, among the students who consumed alcohol a high percentage of both male (31.2%) and female (42.5%) students had problems related to the use of the substance. Moreover, 55.5% of males and 43.7% of females prefer a binge-drinking (BD) pattern.

Our data on BD matched up with those reported by different national and international studies [39,40] that showed how binge drinking has become a common practice aimed at achieving a rapid state of drunkenness, motivated by peer approval and recognition [41,42]. Interestingly, a global survey reported that more than 27% percent of all 15- to 19-year-olds consumed alcohol: in Europe this figure was 44%, in the Americas it was 38%, and in the Western Pacific the figure was 38% [43]. On the other hand, some studies show that ethnicity was a factor to be taken into account. Indeed, white adolescents engaged in more binge drinking at all levels than black, hispanic, or other adolescents of other races/ethnicities [44].

Nonetheless, determinants of heavy alcohol drinking—i.e., legal frameworks, social norms, and culture—vary across the world [45,46]. For instance, whilst the European Mediterranean style of drinking is characterized by daily drinking of wine with meals and no acceptance of public drunkenness, the Northern European pattern relies on drinking spirits in a non-daily pattern and the acceptance of drunkenness in public [47].

Moreover, the adolescents’ motivations for this type of drinking are often instructed, not only by curiosity about experiencing the effects of alcohol or simply “Cheers” with friends, but also by avoidance of events that are often perceived as negative [41]. This can result in engaging in “risky” behaviors that are harmful to oneself and others, such as drunk driving, traffic accidents, risky sexual behavior, and violent or criminal behavior [48,49].

Clinically, BD can lead to premature dysfunction of the cardiovascular and gastrointestinal systems and alter normal brain development [50,51,52]. Notably, it should be mentioned that adolescence is a period of life characterized by continuous dynamic changes in brain structures, occurring in the context of important physiological, psychological, and social transitions [53]. During this period, an asynchronous maturation between the cerebral cortex and limbic system makes the brain more vulnerable to environmental influences, predisposing the adolescent to risky behaviors such as taking different substances of abuse [54,55]. To this end, the brain is sensitive to the effects of alcohol, or its first metabolite acetaldehyde [56], and repeated periods of abstinence, due to alternating periods of alcohol intoxication and sobriety, can impair cognitive function and promote the genesis of different psychiatric disorders, such as anxiety, depression, and straining behavior [57,58,59], which, in turn, influence how alcohol or other substances of abuse are taken and/or consumed [60,61].

In addition to alcohol, nicotine also increases the susceptibility to the use of other substances of abuse [62,63]. For this reason, investigating the number of students who smoke and analyzing their degree of dependence towards the substance was another goal of our study. The results obtained through the administration of the FTND highlighted that a fair percentage of participants who consumed nicotine, with a prevalence of male ones (41.5%) compared to female ones (28%). In addition, the “degree of dependence” analysis revealed a severe degree of dependence (43%) among males, as opposed to females, who showed a slight (37%) rather than moderate (31.5%) degree of dependence towards the substance.

These data, although too limited in number to lead to firm conclusions, are in agreement with other studies [64,65] that show how belonging to either sex can influence nicotine use. Recent data conducted in the Italian student population (15–19 years old) by ESPAD^®^ Italia, reported a reversal in the consumption of at least one cigarette per day. In particular, while until 2008 female students showed a higher prevalence than male students, in subsequent surveys they were overtaken by males [66]. The observed differences in consumption and degree of dependence may be due to unequal genetic makeup [67]. Indeed, linkage studies have identified genes and regions of the genome that significantly influence both treatment efficacy [67,68] and the onset of nicotine dependence [68,69].

Some of the genes that influence the onset and persistence of substance use include those associated with differences in nicotine-metabolizing capacity and expression of its receptors [70,71].

In particular, the hepatic cytochrome P450 (CYP) isoform 2A6 enzyme system appears to play a key role [70]. CYP2A6 genetic variants have been associated with a slower metabolism of nicotine resulting in a higher blood concentration of nicotine [72,73]. This, synergistically with an increased expression of nicotine receptors [74], would promote the risk of developing an addiction [75].

Similarly, nicotine and alcohol increase AMPA and NMDA receptor protein levels [76,77] prompting a hyperglutamatergic state that contributes to the development and expression of dependence [78].

When we analyzed the possible link between nicotine and alcohol use, a clear correlation betweem the use of the two substances in both male and female students was found. These data tied in with several studies reporting the reciprocal influence between the two substances [79]. In addition, patients who were diagnosed with an addiction to alcohol or nicotine simultaneously exhibited an addiction to the other substance [80,81]. This synergism could be traced to a direct action, by nicotine, on its own nicotine acetylcholine receptor sites (nAChRs) and an indirect action on the GABAergic and endorphinergic systems by alcohol. This induces an increase in dopamine signaling in the mesolimbic circuits [82,83,84], thus enhancing the hedonic value of substances of abuse.

These effects are particularly evident during adolescence, since, during this period, brain development is not yet complete and there is not enough effective communication between different brain regions to enable decision making by weighing emotions and reasons. The uncompleted maturation of frontal top-down inhibitory control is not able to curb the activity increase in sub-cortical structure [85,86].

Furthermore, in today’s highly stimulating socio-cultural environment, where skill development, for example, through school, music, verbal communication, and social interaction [87], is needed, an increase in risk-taking behaviors, such as those that drive substance use or abuse, would lead to potentially harmful effects.

## 5. Study Limitations

Although this study provided further clarification regarding modes and amounts of alcohol and nicotine consumed among adolescents, it had several limitations. The sample analyzed was not large enough to lead to definite and unequivocal conclusions, although the results were in line with other observational studies. Due to the health emergency caused by COVID-19 it was not possible to proceed with the evaluation of parameters that were useful for understanding the changes in neurochemical and behavioral structure that underlie the use and abuse of substances analyzed in this study.

## 6. Conclusions

This observational study provided additional insight into the problem of the consumption of “legal” substances of abuse, most commonly used in adolescence. Fully understanding the mechanisms and modalities underlying the use of nicotine and alcohol, and the possible correlation of their simultaneous use, could provide a valuable and effective tool for understanding the neuro-psychological substrate underlying the establishment of the phenomenon of addiction at a young age.

The behavioral factors that predispose the use of substances such as nicotine and alcohol are directly responsible for the prevalent pathologies of our times and for the present and future state of health. Our scientific research aimed to contribute to the response for the need to nurture the wellbeing of youth populations (pre-adolescent and adolescent) whose lack of satisfaction shows up in behaviors that are harmful to health, when not clearly deviant and antisocial. An understanding of the determinants of these risk behaviors and, specularly, of the elaboration of positive values, could thus contribute to the promotion and implementation of social policies.

The adoption of prevention policies aimed at the implementation of specific school training programs could therefore be useful to encourage the adoption of behaviors oriented to a correct psycho-behavioral development that exclude the establishment and maintenance of pathological dependencies.

Further research should consider enrolling more adolescents to understand how variables such as lifestyle, socioeconomic conditions, and history may influence the approach to drug use.

## Figures and Tables

**Table 1:** Demographic data and other baseline characteristic of students included in the study, divided by sex. na: number; %: percentage; mean: mean value; SD: standard deviation; Range Min, Med and Max: respectively, minimum, median, and maximum.

Male	na	%	Mean	SD	Min	Med	Max
Students	224	100					
Age			16.83	1.295	14	17	19
Height (cm)			1.73	0.0768	1.50	1.73	1.97
Weight (kg)			67.37	11.89	40	65	98
Body Mass Index (BMI)							
Under weight	17	7.6	17.39	1.198	14.87	17.99	18.37
Normal weight	164	73.2	21.62	1.855	18.64	21.22	24.82
Overweight	30	13.4	27.26	1.307	25.14	27.45	29.39
Obese	13	5.8	34.49	7.474	30.12	31.22	51.20
							
**Female**	**na**	**%**	**Mean**	**SD**	**Min**	**Med**	**Max**
Students	125	100					
Age			15.82	1.240	14	15	19
Height (cm)			1.64	0.0766	1.43	1.61	1.80
Weight (kg)			55.79	9.33	40	56	78
Body Mass Index (BMI)							
Under weight	31	24.8	16.88	0.966	15.63	16.61	18.38
Normal weight	83	66.4	21.63	2.04	18.59	21.37	29.05
Overweight	11	8.8	26.72	1.758	25.06	25.86	28.91
Obese	0	-	-	-	-	-	

**Table 2:** Data on alcohol consumption of the 224 male and 125 female recruited students’ population. All data were calculated from the results obtained from analysis of AUDIT-C. *n* = number; % = percentage; AUDIT-C = Alcohol Use Disorders Identification Test-Concise; CI = confidence interval.

Male	*n*	%	AUDIT-C Score Index Mean	95% CI
	224	100	2.652	2.368–2.936
Non drinkers	51	22.8	-	-
Drinkers	173	77.2	3.497	3.206–3.788
Among students reporting alcohol intake				
Occasional drinkers	48	27.7	1.604	1.319–1.890
Monthly drinkers	65	37.6	3.185	2.878–3.491
Weekly drinkers	60	34.7	5.133	4.827–5.440
Harmful drinkers	54	31.2	5.648	5.463–5.833
Binging drinkers	96	55.5	4.688	4.421–4.954
**Female**	***n***	**%**	**AUDIT-C Score** **Index Mean**	**95% CI**
	125	100	1.832	1.520–2.144
Non drinkers	38	30.4	-	-
Drinkers	87	69.6	2.793	2.412–3.174
Among students reporting alcohol intake				
Occasional drinkers	37	42.5	1.432	1.165–1.699
Monthly drinkers	30	34.5	3.933	2.542–3.325
Weekly drinkers	20	23.0	4.400	3.824–4.976
Harmful drinkers	39	42.5	4.000	3.676–4.324
Binging drinkers	38	43.7	3.947	3.543–4.351

**Table 3:** Data on nicotine dependence of the 224 male and 125 female recruited students’ population. All data were calculated from the results obtained from analysis of FTND. *n* = number; % = percentage; FTND = Fagerström Test for Nicotine Dependence; CI = confidence interval.

Male	*n*	%	FTND Score Index Mean	95% CI
	224	100	1.612	1.318–1.905
Smoking habit				
Non smokers	131	58.5	-	-
Smokers	93	41.5	3.882	3.519–4.245
Degree of dependence				
Mild	21	22.6	1.429	0.860–1.997
Medium	30	32.2	3.633	3.450–3.816
Severe	40	43.0	5.275	5.130–5.420
Very severe	2	2.2	7.500	1.147–13.85
**Female**	***n***	**%**	**FTND Score** **Index Mean**	**95% CI**
	125	100	0.9200	0.590–1.249
Smoking habit				
Non smokers	90	72	-	-
Smokers	35	28	3.286	2.547–4.024
Degree of dependence				
Mild	13	37.1	1.000	0.4484–1.552
Medium	11	31.5	3.455	3.104–3.805
Severe	9	25.7	5.556	5.150–5.961
Very severe	2	5.7	7.000	7.000–7.000

## Data Availability

The data is not publicly available due to privacy restrictions.

## References

[1] World Health Organization (2018). Harmful Use of Alcohol Kills More than 3 Million People Each Year, Most of Them Men. www.who.int.

[2] Grant M. (1985). Alcohol Policies. Copenhagen: WHO Regional Publications European Series 18.

[3] Gual A., Colom J. (1997). Why has alcohol consumption declined in countries of southern Europe?. Addiction.

[4] World Health Organization (2011). Global Information System on Alcohol and Health.

[5] Istituto Nazionale di Statistica (2020). Il Consumo di Alcol in Italia. www.istat.it/archivio/244222.

[6] Pollard M.S., Tucker J.S., Green H.D. (2020). Changes in Adult Alcohol Use and Consequences During the COVID-19 Pandemic in the US. JAMA.

[7] Cirrincione L., Rapisarda V., Mazzucco W., Provenzano R., Cannizzaro E. (2021). SARS-CoV-2 and the Risk Assessment Document in Italian Work; Specific or Generic Risk Even If Aggravated?. Int. J. Environ. Res. Public Health.

[8] Cirrincione L., Plescia F., Ledda C., Rapisarda V., Martorana D., Lacca G., Argo A., Zerbo S., Vitale E., Vinnikov D. (2022). COVID-19 Pandemic: New Prevention and Protection Measures. Sustainability.

[9] Wang Y., Di Y., Ye J., Wei W. (2021). Study on the public psychological states and its related factors during the outbreak of coronavirus disease 2019 (COVID-19) in some regions of China. Psychol. Health Med..

[10] Cannizzaro E., Cirrincione L., Mazzucco W., Scorciapino A., Catalano C., Ramaci T., Ledda C., Plescia F. (2020). Night-Time Shift Work and Related Stress Responses: A Study on Security Guards. Int. J. Environ. Res. Public Health.

[11] Boscarino J.A., Adams R.E., Galea S. (2006). Alcohol use in New York after the terrorist attacks: A study of the effects of psychological trauma on drinking behavior. Addict. Behav..

[12] Brancato A., Lavanco G., Cavallaro A., Plescia F., Cannizzaro C. (2017). Acetaldehyde, Motivation and Stress: Behavioral Evidence of an Addictive ménage à trois. Front. Behav. Neurosci..

[13] Plescia F., Cirrincione L., Martorana D., Ledda C., Rapisarda V., Castelli V., Martines F., Vinnikov D., Cannizzaro E. (2021). Alcohol Abuse and Insomnia Disorder: Focus on a Group of Night and Day Workers. Int. J. Environ. Res. Public Health.

[14] Kuntsche E., Rehm J., Gmel G. (2004). Characteristics of binge drinkers in Europe. Soc. Sci. Med..

[15] NIAAA Drinking Levels Defined. https://www.niaaa.nih.gov/alcohol-health/overview-alcohol-consumption/moderate-binge-drinking.

[16] Windle M. (2003). Alcohol use among adolescents and young adults. Alcohol Res. Health.

[17] Waller R., Murray L., Shaw D.S., Forbes E.E., Hyde L.W. (2019). Accelerated alcohol use across adolescence predicts early adult symptoms of alcohol use disorder via reward-related neural function. Psychol. Med..

[18] Van Roy B., Veenstra M., Clench-Aas J. (2008). Construct validity of the five-factor Strengths and Difficulties Questionnaire (SDQ) in pre-, early, and late adolescence. J. Child Psychol. Psychiatry.

[19] Kuntsche E., Gmel G. (2013). Alcohol consumption in late adolescence and early adulthood—Where is the problem?. Swiss Med. Wkly..

[20] Santangelo O.E., Provenzano S., Piazza D., Firenze A. (2018). Factors associated with risky con-sumption of alcohol in a sample of university students. Ann. Ig..

[21] Briones T.L., Woods J. (2013). Chronic binge-like alcohol consumption in adolescence causes depression-like symptoms possibly mediated by the effects of BDNF on neurogenesis. Neuroscience.

[22] McBride O., Cheng H.G. (2011). Exploring the emergence of alcohol use disorder symptoms in the two years after onset of drinking: Findings from the National Surveys on drug use and health. Addiction.

[23] Golub A., Johnson B.D. (1994). The shifting importance of alcohol and marijuana as gateway substances among serious drug abusers. J. Stud. Alcohol.

[24] Davila V.R., Stahl D.L., Bhandary S.P., Papadimos T.J. (2018). What’s New in Critical Illness and Injury Science? The association between initial blood alcohol concentration and polysubstance use may be indicative of a gateway drug effect. Int. J. Crit. Illn. Inj. Sci..

[25] Nunes P.T., Kipp B.T., Reitz N.L., Savage L.M. (2019). Aging with alcohol-related brain damage: Critical brain circuits associated with cognitive dysfunction. Int. Rev. Neurobiol..

[26] Sartor C.E., Lynskey M.T., Heath A.C., Jacob T., True W. (2007). The role of childhood risk factors in initiation of alcohol use and progression to alcohol dependence. Addiction.

[27] Brancato A., Castelli V., Lavanco G., Tringali G., Micale V., Kuchar M., D’Amico C., Pizzolanti G., Feo S., Cannizzaro C. (2021). Binge-like Alcohol Exposure in Adolescence: Behavioural, Neuroendocrine and Molecular Evidence of Abnormal Neuroplasticity and Return. Biomedicines.

[28] Tapia-Rojas C., Carvajal F.J., Mira R.G., Arce C., Lerma-Cabrera J.M., Orellana J.A., Cerpa W., Quintanilla R.A. (2018). Adolescent Binge Alcohol Exposure Affects the Brain Function Through Mitochondrial Impairment. Mol. Neurobiol..

[29] West R.K., Maynard M.E., Leasure J.L. (2018). Binge ethanol effects on prefrontal cortex neurons, spatial working memory and task-induced neuronal activation in male and female rats. Physiol. Behav..

[30] Voos N., Goniewicz M.L., Eissenberg T. (2019). What is the nicotine delivery profile of electronic cigarettes?. Expert Opin. Drug Deliv..

[31] Jordt S.E. (2021). Synthetic nicotine has arrived. Tob. Control.

[32] Istituto Superiore di Sanità Fumo Informazioni Generali. www.epicentro.iss.it/fumo/.

[33] Torres O.V., Tejeda H.A., Natividad L.A., O’Dell L.E. (2008). Enhanced vulnerability to the rewarding effects of nicotine during the adolescent period of development. Pharmacol. Biochem. Behav..

[34] Colby S.M., Tiffany S.T., Shiffman S., Niaura R.S. (2000). Are adolescent smokers dependent on nicotine? A review of the evidence. Drug Alcohol Depend..

[35] Coulton S., Alam M.F., Boniface S., Deluca P., Donoghue K., Gilvarry E., Kaner E., Lynch E., Maconochie I., McArdle P. (2019). Opportunistic screening for alcohol use problems in adolescents attending emergency departments: An evaluation of screening tools. J. Public Health.

[36] Caputo A. (2020). Comparing Theoretical Models for the Understanding of Health-Risk Behaviour: Towards an Integrative Model of Adolescent Alcohol Consumption. Eur. J. Psychol..

[37] Chen X., Zheng H., Steve S., Gong J., Stacy A., Xia J., Gallaher P., Dent C., Azen S., Shan J. (2002). Use of the fagerstrom tolerance questionnaire for measuring nicotine dependence among adolescent smokers in China: A pilot test. Psychol. Addict. Behav..

[38] Fekketich A.K., Fossati R., Apolone G. (2008). An Evaluation of the Italian Version of the Fagerström Test for Nicotine Dependence. Psychol. Rep..

[39] Patrick M.E., Schulenberg J.E., Martz M.E., Maggs J.L., O’Malley P.M., Johnston L.D. (2013). Extreme binge drinking among 12th-grade students in the United States: Prevalence and predictors. JAMA Pediatr..

[40] Martinotti G., Lupi M., Carlucci L., Santacroce R., Cinosi E., Acciavatti T., Sarchione F., Verrastro V., Diotaiuti P., Petruccelli I. (2017). Alcohol drinking patterns in young people: A survey-based study. J. Health Psychol..

[41] Kuntsche E., Müller S. (2012). Why do young people start drinking? Motives for first-time alcohol consumption and links to risky drinking in early adolescence. Eur. Addict. Res..

[42] Chung T., Creswell K.G., Bachrach R., Clark D.B., Martin C.S. (2018). Adolescent Binge Drinking. Alcohol Res..

[43] Alcol e Salute, la Situazione Globale nel Report Oms (iss.it). www.epicentro.iss.it/alcol/GlobalStatusReportAlcol2018.

[44] Chassin L., Pitts S.C., Prost J. (2002). Binge drinking trajectories from adolescence to emerging adulthood in a high-risk sample: Predictors and substance abuse outcomes. J. Consult Clin. Psychol..

[45] Sudhinaraset M., Wigglesworth C., Takeuchi D.T. (2016). Social and Cultural Contexts of Alcohol Use: Influences in a Social-Ecological Framework. Alcohol Res..

[46] Halim A., Hasking P., Allen F. (2012). The role of social drinking motives in the relationship between social norms and alcohol consumption. Addict. Behav..

[47] Popova S., Rehm J., Patra J., Zatonski W. (2007). Comparing alcohol consumption in central and eastern Europe to other European countries. Alcohol Alcohol..

[48] Miller J.W., Naimi T.S., Brewer R.D., Jones S.E. (2007). Binge drinking and associated health risk behaviors among high school students. Pediatrics.

[49] Dube S.R., Miller J.W., Brown D.W., Giles W.H., Felitti V.J., Dong M., Anda R.F. (2006). Adverse childhood experiences and the association with ever using alcohol and initiating alcohol use during adolescence. J. Adolesc. Health.

[50] Paus T., Zijdenbos A., Worsley K., Collins D.L., Blumenthal J., Giedd J.N., Rapoport J.L., Evans A.C. (1999). Structural maturation of neural pathways in children and adolescents: In vivo study. Science.

[51] Day E., Rudd J.H.F. (2019). Alcohol use disorders and the heart. Addiction.

[52] Shimpi T.R., Shikhare S.N., Chung R., Wu P., Peh W.C.G. (2019). Imaging of Gastrointestinal and Abdominal Emergencies in Binge Drinking. Can. Assoc. Radiol. J..

[53] Bava S., Tapert S.F. (2010). Adolescent brain development and the risk for alcohol and other drug problems. Neuropsychol. Rev..

[54] De Luca C.R., Wood S.J., Anderson V., Buchanan J.A., Proffitt T.M., Mahony K., Pantelis C. (2003). Normative data from the CANTAB. I: Development of executive function over the lifespan. J. Clin. Exp. Neuropsychol..

[55] Clark D.B., Thatcher D.L., Tapert S.F. (2008). Alcohol, psychological dysregulation, and adolescent brain development. Alcohol Clin. Exp. Res..

[56] Plescia F., Brancato A., Venniro M., Maniaci G., Cannizzaro E., Sutera F.M., De Caro V., Giannola L.I., Cannizzaro C. (2015). Acetaldehyde self-administration by a two-bottle choice paradigm: Consequences on emotional reactivity, spatial learning, and memory. Alcohol.

[57] Cranford J.A., Eisenberg D., Serras A.M. (2009). Substance use behaviors, mental health problems, and use of mental health services in a probability sample of college students. Addict. Behav..

[58] Zeng R., Wang L., Xie Y. (2016). An analysis of factors influencing drinking relapse among patients with alcohol-induced psychiatric and behavioral disorders. Shanghai Arch. Psychiatry.

[59] Sutera F.M., De Caro V., Cannizzaro C., Giannola L.I., Lavanco G., Plescia F. (2016). Effects of DA-Phen, a dopamine-aminoacidic conjugate, on alcohol intake and forced abstinence. Behav. Brain Res..

[60] Sullivan L.E., Fiellin D.A., O’Connor P.G. (2005). The prevalence and impact of alcohol problems in major depression: A systematic review. Am. J. Med..

[61] Robinson J., Sareen J., Cox B., Bolton J. (2011). Role of Self-medication in the Development of Comorbid Anxiety and Substance Use Disorders: A Longitudinal Investigation. Arch. Gen. Psychiatry.

[62] Degenhardt L., Dierker L., Chiu W.T., Medina-Mora M.E., Neumark Y., Sampson N., Alonso J., Angermeyer M., Anthony J.C., Bruffaerts R. (2010). Evaluating the drug use “gateway” theory using cross-national data: Consistency and associations of the order of initiation of drug use among participants in the WHO World Mental Health Surveys. Drug Alcohol Depend..

[63] Samet J.M. (2013). Tobacco smoking: The leading cause of preventable disease worldwide. Thorac. Surg. Clin..

[64] Sansone N., Fong G.T., Lee W.B., Laux F.L., Sirirassamee B., Seo H.G., Omar M., Jiang Y. (2013). Comparing the experience of regret and its predictors among smokers in four asian countries: Findings from the itc surveys in Tailand, South Korea, Malaysia, and China. Nicotine Tob. Res..

[65] Alotaibi S.A., Alsuliman M.A., Durgampudi P.K. (2019). Smoking tobacco prevalence among college students in the Kingdom of Saudi Arabia: Systematic review and meta-analysis. Tob. Induc. Dis..

[66] ESPAD ITALIA European School Survey Project on Alcohol and other Drugs—Italy. www.espad.it.

[67] Becker J.B., McClellan M.L., Reed B.G. (2017). Sex differences, gender and addiction. J. Neurosci. Res..

[68] Gelernter J., Panhuysen C., Weiss R., Brady K., Poling J., Krauthammer M. (2007). Genomewide linkage scan for nicotine dependence: Identification of a chromosome 5 risk locus. Biol. Psychiatry.

[69] Bierut L.J., Madden P.A., Breslau N., Johnson E.O., Hatsukami D., Pomerleau O.F. (2007). Novel genes identified in a high-density genome wide association study for nicotine dependence. Hum. Mol. Genet..

[70] Ring H.Z., Valdes A.M., Nishita D.M., Prasad S., Jacob P., Tyndale R.F., Swan G.E., Benowitz N.L. (2007). Gene-gene interactions between CYP2B6 and CYP2A6 in nicotine metabolism. Pharm. Genom..

[71] Bierut L.J., Stitzel J.A., Wang J.C., Hinrichs A.L., Grucza R.A., Xuei X., Saccone N.L., Saccone S.F., Bertelsen S., Fox L. (2008). Variants in nicotinic receptors and risk for nicotine dependence. Am. J. Psychiatry.

[72] Oscarson M. (2001). Genetic polymorphisms in the cytochrome P450 2A6 (CYP2A6) gene: Implications for interindividual differences in nicotine metabolism. Drug Metab. Dispos..

[73] Yoshida R., Nakajima M., Nishimura K., Tokudome S., Kwon J.T., Yokoi T. (2003). Effects of polymorphism in promoter region of human CYP2A6 gene (CYP2A6*9) on expression level of messenger ribonucleic acid and enzymatic activity in vivo and in vitro. Clin. Pharmacol. Ther..

[74] Colombo S.F., Mazzo F., Pistillo F., Gotti C. (2013). Biogenesis, trafficking and up-regulation of nicotinic ACh receptors. Biochem. Pharmacol..

[75] Karp I., O’Loughlin J., Hanley J., Tyndale R.F., Paradis G. (2006). Risk factors for tobacco dependence in adolescent smokers. Tob. Control.

[76] Wang F., Chen H., Steketee J.D., Sharp B.M. (2007). Upregulation of ionotropic glutamate receptor subunits within specific mesocorticolimbic regions during chronic nicotine self-administration. Neuropsychopharmacol.

[77] Szumlinski K.K., Woodward J.J. (2014). Glutamate signaling in alcohol abuse and dependence. Neurobiol Alcohol Depend..

[78] Castelli V., Brancato A., Cavallaro A., Lavanco G., Cannizzaro C. (2017). Homer2 and Alcohol: A Mutual Interaction. Front. Psychiatry.

[79] Verplaetse T.L., McKee S.A. (2017). An overview of alcohol and tobacco/nicotine interactions in the human laboratory. Am. J. Drug Alcohol Abus..

[80] McKee S.A., Falba T., O’Malley S.S., Sindelar J., O’Connor P.G. (2007). Smoking status as a clinical indicator for alcohol misuse in US adults. Arch. Intern. Med..

[81] Grant B.F. (1998). Age at smoking onset and its association with alcohol consumption and DSM-IV alcohol abuse and dependence: Results from the National Longitudinal Alcohol Epidemiologic Survey. J. Subst. Abus..

[82] Di Chiara G. (2000). Role of dopamine in the behavioural actions of nicotine related to addiction. Eur. J. Pharmacol..

[83] Mansvelder H.D., McGehee D.S. (2000). Long-term potentiation of excitatory inputs to brain reward areas by nicotine. Neuron.

[84] Miyata H., Yanagita T. (2001). Neurobiological mechanisms of nicotine craving. Alcohol.

[85] Gogtay N., Giedd J.N., Lusk L., Hayashi K.M., Greenstein D., Vaituzis A.C., Nugent T.F., Herman D.H., Clasen L.S., Toga A.W. (2004). Dynamic mapping of human cortical development during childhood through early adulthood. Proc. Natl. Acad. Sci. USA.

[86] Casey B., Jones R.M., Somerville L.H. (2011). Braking and accelerating of the adolescent brain. J. Res. Adolesc..

[87] Singer W. (2011). Dynamic formation of functional networks by synchronization. Neuron.

